# Feasibility of Different Tumor Delineation Approaches for ^18^F-PSMA-1007 PET/CT Imaging in Prostate Cancer Patients

**DOI:** 10.3389/fonc.2021.663631

**Published:** 2021-05-21

**Authors:** Lena M. Mittlmeier, Matthias Brendel, Leonie Beyer, Nathalie L. Albert, Andrei Todica, Mathias J. Zacherl, Vera Wenter, Annika Herlemann, Alexander Kretschmer, Stephan T. Ledderose, Nina-Sophie Schmidt-Hegemann, Wolfgang G. Kunz, Jens Ricke, Peter Bartenstein, Harun Ilhan, Marcus Unterrainer

**Affiliations:** ^1^ Department of Nuclear Medicine, University Hospital, Ludwig Maximilian University (LMU) Munich, Munich, Germany; ^2^ Department of Urology, University Hospital, LMU Munich, Munich, Germany; ^3^ Department of Pathology, University Hospital, LMU Munich, Munich, Germany; ^4^ Department of Radiation Oncology, University Hospital, LMU Munich, Munich, Germany; ^5^ Department of Radiology, University Hospital, LMU Munich, Munich, Germany

**Keywords:** PSMA, PET, mCRPC, Metastatic castrate-resistant prostate cancer, prostate cancer, whole tumor volume

## Abstract

**Background:**

Delineation of PSMA-positive tumor volume on PET using PSMA-ligands is of highest clinical interest as changes of PSMA-PET/CT-derived whole tumor volume (WTV) have shown to correlate with treatment response in metastatic prostate cancer patients. So far, WTV estimation was performed on PET using ^68^Ga-labeled ligands; nonetheless, ^18^F-labeled PET ligands are gaining increasing importance due to advantages over ^68^Ga-labeled compounds. However, standardized tumor delineation methods for ^18^F-labeled PET ligands have not been established so far. As correlation of PET-based information and morphological extent in osseous and visceral metastases is hampered by morphological delineation, low contrast in liver tissue and movement artefacts, we correlated CT-based volume of lymph node metastases (LNM) and different PET-based delineation approaches for thresholding on ^18^F-PSMA-1007 PET.

**Methods:**

Fifty patients with metastatic prostate cancer, ^18^F-PSMA-1007 PET/CT and non-bulky LNM (short-axis diameter ≥10mm) were included. Fifty LNM were volumetrically assessed on contrast-enhanced CT (volumetric reference standard). Different approaches for tumor volume delineation were applied and correlated with the reference standard: I) fixed SUV threshold, II) isocontour thresholding relative to SUV_max_ (SUV%), and thresholds relative to III) liver (SUV_liver_), IV) parotis (SUV_parotis_) and V) spleen (SUV_spleen_).

**Results:**

A fixed SUV of 4.0 (r=0.807, r^2^ = 0.651, p<0.001) showed the best overall association with the volumetric reference. 55% SUV_max_ (r=0.627, r^2^ = 0.393, p<0.001) showed highest association using an isocontour-based threshold. Best background-based approaches were 60% SUV_liver_ (r=0.715, r^2^ = 0.511, p<0.001), 80% SUV_parotis_ (r=0.762, r^2^ = 0.581, p<0.001) and 60% SUV_spleen_ (r=0.645, r^2^ = 0.416, p<0.001). Background tissues SUV_liver,_ SUV_parotis_ & SUV_spleen_ did not correlate (p>0.05 each). Recently reported cut-offs for intraprostatic tumor delineation (isocontour 44% SUV_max_, 42% SUV_max_ and 20% SUV_max_) revealed inferior association for LNM delineation.

**Conclusions:**

A threshold of SUV 4.0 for tumor delineation showed highest association with volumetric reference standard irrespective of potential changes in PSMA-avidity of background tissues (e. g. parotis). This approach is easily applicable in clinical routine without specific software requirements. Further studies applying this approach for total tumor volume delineation are initiated.

## Introduction

Prostate-specific membrane antigen (PSMA) targeted positron-emission-tomography (PET)/computed tomography (CT) is increasingly used for prostate cancer (PCa) staging and localization of recurrent and/or advanced disease (1). International PCa guidelines, including the European Association of Urology guideline, recommend PSMA PET/CT and its use, specifically in patients with PSA recurrence after primary therapy. Recently, the proPSMA trial also highlighted the important role of PSMA PET in high-risk patients prior to curative-intent surgery or radiotherapy with superior accuracy and lower costs compared to conventional imaging (2, 3). Furthermore, PCa staging using PSMA PET has significant impact on patient management as demonstrated in several groups (1, 4–8).

Beyond staging, PSMA PET/CT represents a useful tool for response to systemic therapy such as chemotherapy and radioligand therapy using ^177^Lu-PSMA ligands (1, 9, 10). Here, PSMA PET/CT provides additional information beyond the most commonly used tools for oncological response assessment in clinical trials such as CT, magnetic resonance imaging (MRI), bone scintigraphy and PSA serum levels (9, 11–13). Due to the limited diagnostic and predictive accuracy of morphological criteria, such as Response Evaluation Criteria in Solid Tumors (RECIST), particularly in mCRPC patients, advanced imaging-based response assessment tools with higher accuracy are needed, like it is the case with ^18^F-FDG-PET/CT in other tumor types like non-small-cell lung cancer (9, 14–16).

In this context, the longitudinal course of the PET-derived whole tumor volume (WTV) during systemic therapies is gaining increasing interest as an additional imaging biomarker for therapy monitoring. Several studies demonstrated that changes of PSMA PET-derived WTV correlate with treatment response (1, 9, 11, 17, 18) and may also serve as prognostic tool for overall survival estimation (1, 19, 20), as recently highlighted by a consensus statement by Fanti et al. (1).

In the field of PSMA ligands, ^18^F-labeled PSMA ligands will become increasingly important due to their advantages compared to ^68^Ga-labeled compounds, e. g. longer half-life, a lower positron energy and the possibility of large-batch production (21). While there are already published studies for tracer-specific thresholding and window-level-setting for WTV delineation using ^68^Ga-labeled ligands, to the best of our knowledge no study so far evaluated different models for WTV estimation using ^18^F-labeled PSMA ligands hitherto. So far, only two studies focused on intraprostatic tumor delineation using ^18^F-PSMA-1007, but without application to WTV (21, 22). Hence, we aimed at identifying and comparing different thresholding approaches for tumor delineation on ^18^F-PSMA-1007 PET/CT in correlation to a direct, CT-based volumetric reference standard.

Even if bone metastases present a common and clinically relevant metastatic spread in PCa patients (23), they are difficult to delineate on CT, mostly deeming them as non-measurable lesions according to RECIST 1.1 (24, 25). Also, lung metastases represent an unideal reference standard, especially due to motion artefacts on PET/CT and unequivocal protocols concerning breath-holding impacting PET imaging. In contrast, LNM represent measurable metastatic sites, especially in case of large extent and non-bulky localization. Therefore, we used large, non-bulky lymph nodes as volumetric reference standard for the evaluation of different threshold approaches for tumor delineation on ^18^F-PSMA-1007 PET/CT.

## Material and Methods

### Inclusion Criteria

This retrospective analysis was approved by the institutional ethics committee of the LMU Munich. Criteria for inclusion were I) patients with known or highly suspected (i.e., highly increased PSA value) metastatic prostate cancer; II) ^18^F-PSMA-1007 PET/CT, III) at least one singular located, non-bulky and PSMA-avid lymph node metastasis with short axis diameter (SAD) ≥ 1.0 cm.

### Radiopharmaceutical and Imaging Protocol

A median activity of 247 MBq (range, 192-306 MBq) ^18^F-PSMA-1007 was injected intravenously in line with previously reported radiosynthesis and administration procedures (26). The patients were premedicated with furosemide (20 mg intravenously), when no contraindication was noted (27). The administration of the radiopharmaceutical was based on an individual patient basis according to the German Pharmaceuticals Act §13(2b). PET was performed from skull base to mid-thigh using a Biograph mCT scanner or a Biograph 64 PET/CT scanner (Siemens Healthineers Erlangen, Germany). The PET/CT scan was performed 60 min after tracer injection which included a diagnostic, contrast-enhanced CT scan in portal-venous phase (Imeron 350; 1.5 ml/kg body weight; Bracco Imaging, Milano, Italy). Images were reconstructed iteratively using TrueX (three iterations, 21 subsets) with Gaussian post-reconstruction smoothing (2 mm full width at half-maximum). Slice thickness on contrast-enhanced CT was 0.3 cm.

### CT Image Analysis

For lymph node analysis, the SAD and the long-axis-diameter (LAD) were assessed. Assessment criterion for lymph node metastases were SAD of at least 1.0 cm, non-bulky, singular located and a distinct localization without contact to other structures. The extent of PSMA-avidity was no criterion for the selection of lymph node metastases. Then, the volume of the respective lymph nodes was manually delineated on a slice-by-slice manner and visually checked for correctness. The respective localizations were determined in each of the selected LNM (one per patient) by two experienced radiologists (WGK, MU) on a dedicated workstation (Siemens Healthineers Erlangen, Germany).

### PET Image Analysis

Using a dedicated workstation (Affinity 1.1.4, Hermes Medical Solutions, Stockholm, Sweden) an ellipsoid volume of interest (VOI) was created surrounding the selected lymph node excluding off-target, PSMA-avid lesions. Exclusion of other PSMA-avid lesions was checked visually in order to avoid biased results. In this VOI, different approaches for volumetric delineation of the respective lymph nodes were applied and correlated with the reference standard; the following approaches were used: I) fixed SUV threshold, II) isocontour thresholding relative to SUV_max_ (SUV%) and thresholds relative to III) liver (SUV_liver_), IV) parotis (SUV_parotis_) and V) spleen (SUV_spleen_):

- Fixed SUV thresholds: The following values were applied: SUV 15.0; SUV 10.0; SUV 7.5; SUV 5.0; SUV 4.5; SUV 4; SUV 3.5; SUV 3.0 and SUV 2.5).
- Isocontour relative to SUV_max_ (SUV%): The following values were applied: 10.0%; 15.0%, 20.0%, 25.0%, 30.0%, 35.0%, 40.0%, 42.0%, 44.0%, 45.0%, 50.0%, 55.0%; 50.0%; 70.0% and 75.0%).
- Thresholds relative to SUV_liver_: Background values were derived from a 30 mm-diameter circular reference region of interest (ROI) in the normal inferior right liver lobe in the axial plane excluding blood vessel activity, as described previously (28). The following threshold values were applied: SUV_liver_ minus 45.0%; 50.0%; 55.0%; 60.0%; 70.0% and 75.0%.
- Thresholds relative to SUV_parotis_: Values were derived from a cubic 10 x 10 x 10 mm reference ROI in the parotis. The following threshold values were applied: SUV_parotis_ minus 60.0%; 70.0%; 75.0%; 80.0%; 85.0% and 90.0%.
- Thresholds relative to SUV_spleen_: Background values were derived from a cubic 30 x 30 x 30 mm reference ROI in the spleen. The following threshold values were applied: SUV_spleen_ minus 40.0%; 50.0%; 55.0%; 60.0%; 65.0% and 70.0%.

### Statistical Analyses

Statistical analyses were performed with IBM SPSS^®^ Statistics (version 25, IBM Corp., Armonk, NY). Correlation between CT-measured volumes and the PET-based volumes using different threshold was evaluated using Spearman and Pearson correlation coefficient after testing for normal distribution as determined by the Shapiro-Wilk test. The coefficient of variation (CoV) was used as standardized measure of dispersion of a probability distribution as defined as the ratio of the standard to the mean. Group comparisons of continuous, not normally distributed parameters were compared using the Kruskal-Wallis test. For visualization of correlation, scatter plots and Bland-Altman plots were used. Statistical significance was defined as a two-sided p-value <0.05.

## Results

### Patients

The median age was 71.0 years (range, 55.8-91.5 years). There was a median PSA of 25.8 ng/ml (range, 0.2 – 1118.0 ng/ml) and a median Gleason score of 9 (range, 6 – 10). Lymph node metastases were present in 50/50 patients (100.0%), tumors at the prostate bed in 28/50 patients (56.0%), bone metastases in 36/50 patients (72.0%) and visceral metastases in 11/50 patients (22.0%). Non PSMA-avid metastatic lesions were present in 0/50 patients (0.0%). Extended patients’ specifications including previous therapies are listed in the **Supplementary Table**.

### CT Image Analysis

Lymph node size was assessed using the SAD (median 1.4 cm (range, 1.0 – 2.8 cm), LAD (median 1.9 cm; range 1.1 – 3.8 cm) and CT-derived volume (median 3.2 ml; range 1.0 – 23.8 ml). Among the lymph node metastases, 31/50 were located next to the common and internal iliac vessels (62.0%), 6/50 cervical (12.0%), 3/50 mediastinal (6.0%), 3/50 paraaortic and paracaval/interaortocaval (6.0%), 2/50 in the inguinal region (4.0%), 2/50 pararectal (4.0%), 2/50 axillar (4.0%) and1/50 in the retroclavicular region (2.0%).

### Volumetric Correlation of Different Delineation Approaches

Results from above mentioned I) fixed SUV thresholds, II) isocontour thresholding relative to SUV_max_ (SUV%), thresholds relative to III) liver (SUV_liver_), IV) parotis (SUV_parotis_) and V) spleen (SUV_spleen_) and their correlation to the CT derived volume as reference standard can be found in **Tables 1**–**5**.

- Fixed SUV thresholds: In I) the highest correlation between CT-derived volume and a fixed threshold could be found with a SUV of 4.0 (r=0.807, r^2^ = 0.651, p<0.001). Generally, it could be shown that higher (e. g. 15.0; 10.0), but also lower fixed SUV values (e. g. 2.5, 5.0 and 4.5) comprised lower correlation to the reference standard (please see **Table 1**), due to a consecutive under- and overestimation of the respective volume.
- socontour relative to SUV_max_ (SUV%): 55% SUV_max_ showed highest association using an isocontour (r=0.627, r^2^ = 0.393, p<0.001). Recently reported isocontour based cut-offs for intraprostatic tumor delineation [i. e. isocontour 20%, 44% and 42% SUV_max_ (21, 22)] revealed inferior association for LNM delineation (please see **Table 2**).
- Thresholds relative to SUV_liver_: 60% SUV_liver_ (r=0.715, r^2^ = 0.511, p<0.001) showed highest association using thresholds relative to the SUV_mean_ of the liver while lower as well as higher values relative to the liver showed lower correlation to the reference standard (see **Table 3**).
- Thresholds relative to SUV_parotis_: 80% SUV_parotis_ (r=0.762, r^2^ = 0.581, p<0.001) showed highest association using thresholds relative to the SUV_mean_ of the parotis (SUV_parotis_). Lower values relative to the parotis (e. g. 60% SUV_parotis_), but also higher values (e. g. 90% SUV_parotis_) showed inferior correlation to the volumetric reference standard (see **Table 3**).
- Thresholds relative to SUV_spleen_: 60% SUV_spleen_ (r=0.645, r^2^ = 0.416, p<0.001) showed highest association using thresholds relative to the SUV_mean_ of the spleen (SUV_spleen_). Lower as well as higher threshold values showed lower correlations respectively (see **Table 3**).

**Table 1:** Correlation with fixed SUV thresholds.

Parameter	r-value	r^2^-value	Level of significance
**SUV 15.0**	0.415	0.172	p<0.001
**SUV 10.0**	0.575	0.331	p<0.001
**SUV 7.5**	0.633	0.401	p<0.001
**SUV 5.0**	0.788	0.621	p<0.001
**SUV 4.5**	0.802	0.643	p<0.001
**SUV 4.0**	0.807	0.651	p<0.001
**SUV 3.5**	0.802	0.643	p<0.001
**SUV 3.0**	0.800	0.640	p<0.001
**SUV 2.5**	0.792	0.627	p<0.001

**Table 2:** Isocontour volumetric correlation.

Parameter	r-value	r^2^-value	Level of significance
**Iso 10%**	0.481	0.231	p<0.001
**Iso 15%**	0.440	0.194	p=0.001
**Iso 20%**	0.460	0.212	p<0.001
**Iso 25%**	0.477	0.228	p<0.001
**Iso 30%**	0.520	0.270	p<0.001
**Iso 35%**	0.505	0.255	p<0.001
**Iso 40%**	0.529	0.280	p<0.001
**Iso 42%**	0.530	0.281	p<0.001
**Iso 44%**	0.552	0.305	p<0.001
**Iso 45%**	0.543	0.295	p<0.001
**Iso 50%**	0.604	0.365	p<0.001
**Iso 55%**	0.627	0.393	p<0.001
**Iso 60%**	0.619	0.383	p<0.001
**Iso 65%**	0.610	0.372	p<0.001
**Iso 70%**	0.605	0.366	p<0.001
**Iso 75%**	0.541	0.293	p<0.001

**Table 3:** Background based volumetric correlations with SUV_liver_, SUV_parotis_ and SUV_spleen_.

Parameter	r-value	r^2^-value	Level of significance
**SUV_liver_**			
**45% SUV_liver_**	0.693	0.480	p<0.001
**50% SUV_liver_**	0.693	0.480	p<0.001
**55% SUV_liver_**	0.711	0.506	p<0.001
**60% SUV_liver_**	0.715	0.511	p<0.001
**70% SUV_liver_**	0.690	0.467	p<0.001
**75% SUV_liver_**	0.697	0.486	p<0.001
**SUV_parotis_**			
**60% SUV_parotis_**	0.545	0.297	p<0.001
**70% SUV_parotis_**	0.666	0.444	p<0.001
**75% SUV_parotis_**	0.745	0.555	p<0.001
**80% SUV_parotis_**	0.762	0.581	p<0.001
**85% SUV_parotis_**	0.650	0.423	p<0.001
**90% SUV_parotis_**	0.603	0.364	p<0.001
**SUV_spleen_**			
**40% SUV_spleen_**	0.595	0.354	p<0.001
**50% SUV_spleen_**	0.642	0.412	p<0.001
**55% SUV_spleen_**	0.639	0.408	p<0.001
**60% SUV_spleen_**	0.645	0.412	p<0.001
**65% SUV_spleen_**	0.618	0.382	p<0.001
**70% SUV_spleen_**	0.618	0.382	p<0.001

**Table 4:** Correlation of background tissues SUV_liver,_ SUV_parotis_ & SUV_spleen_.

Parameter	Spleen	Liver	Parotis
**SUV_mean_** [median (range)]	9.9 (4.7 - 28.7)	11.3 (4.2 - 25.5)	20.1 (5.8 - 36.3)
**Coefficient of variation**	42.6%	40.2%	35.6%
**Correlation with spleen**	–	r=0.082 (p=0.572)	r=0.120 (p=0.406)
**Correlation with liver**	r=0.082 (p=0.572)	–	r=0.028 (p=0.845)
**Correlation with parotis**	r=0.120 (p=0.406)	r=0.028 (p=0.845)	–

**Table 5:** Individual backwards thresholding.

	SUV
**Mean ± standard deviation**	5.4 ± 2.4
**Coefficent of variation (CoV)**	44.4%
**Correlation to CT reference (SUV 5.4)**	r=0.764
**Coefficient of determination (SUV 5.4)**	r^2^ = 0.584
**Level of significance (SUV 5.4)**	p<0.001

**Figure 1:**
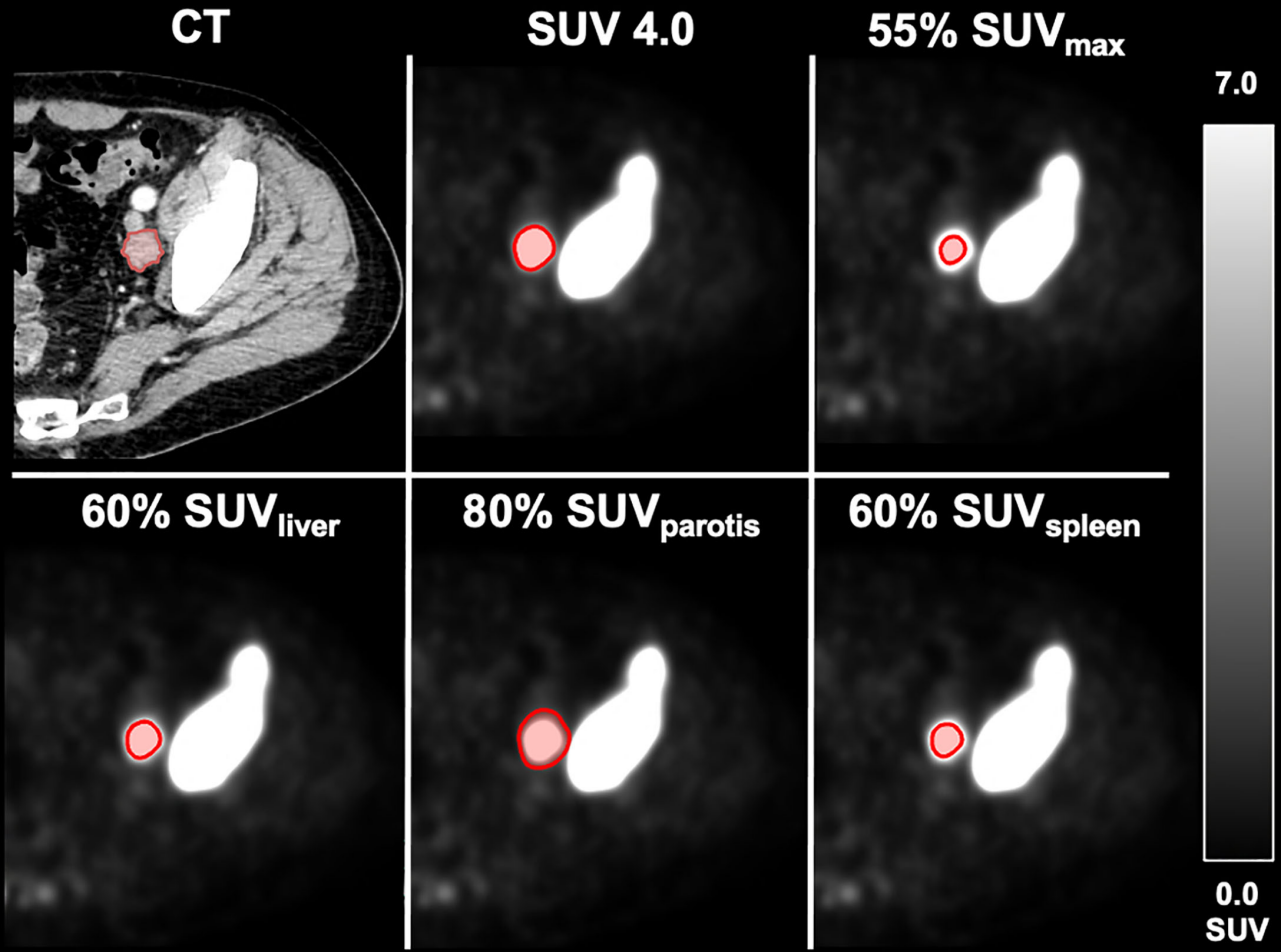
Different delineation methods in an exemplary metastatic patient. Volumetric reference standard 6.3 m; SUV 4.0: 5.5 ml. 55% SUV_max_: 1.0 ml. 60% SUV_liver_: 4.5 ml. 80% SUV_parotis_: 6.4 ml. 60% SUV_spleen_: 4.0 ml.

**Figure 2:**
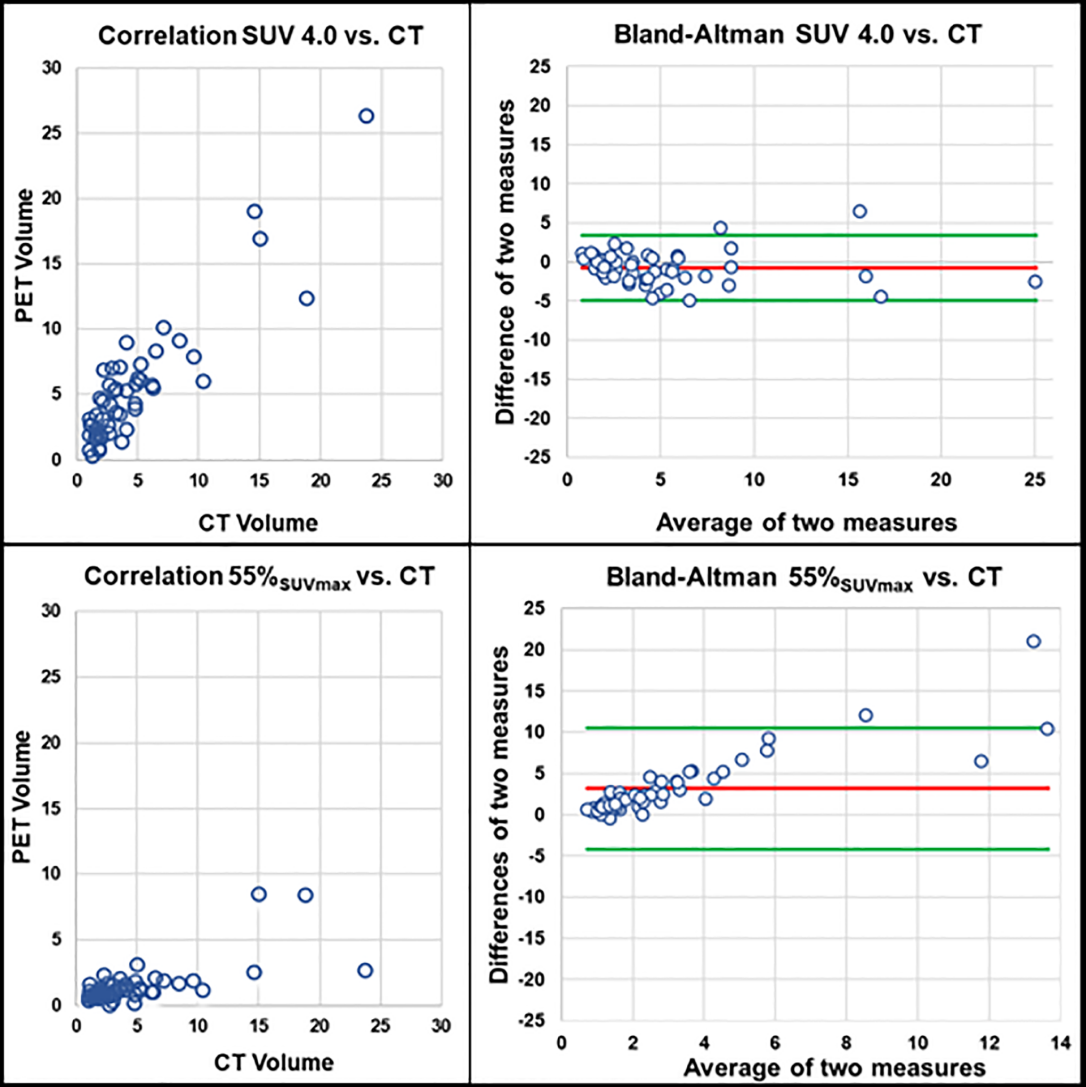
Correlation of PET volumes and CT-based reference standard. Upper row: PET volume SUV 4.0 (r = 0.807, r^2^ = 0.651, p < 0.001). Lower row: PET volume isocontour of 55% SUV_max_ (r = 0.627, r^2^ = 0.393, p < 0.001); each correlation plot is accompanied by the respective Bland-Altman plot (red line: mean difference of two measures. Green lines: mean difference of two measures ± 1.96 x standard deviation).

**Figure 3:**
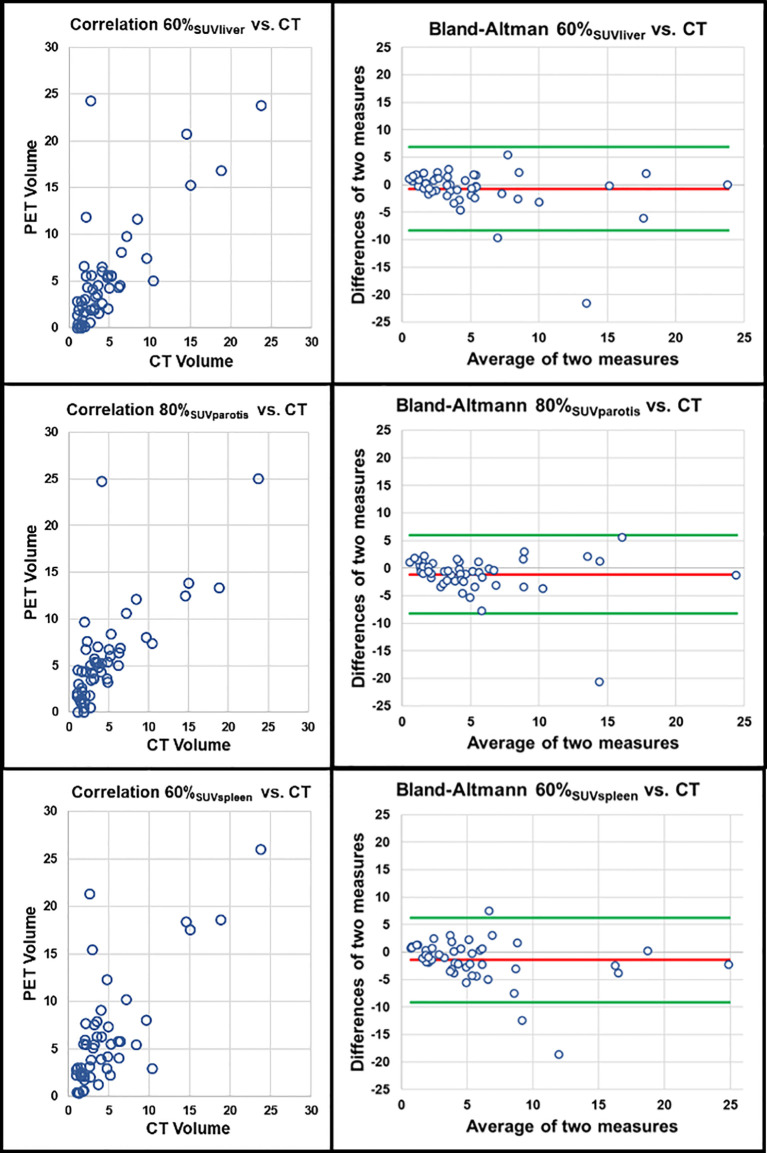
Correlation of PET volumes using background tissue and CT-based reference standard. Upper row: PET volume 60% SUV_liver_ (r = 0.715, r^2^ = 0.511, p < 0.001). Middle row: 80% SUV_parotis_ (r = 0.762, r^2^ = 0.581, p < 0.001). Lower row: PET volume 60% SUV_spleen_ (r = 0.645, r^2^ = 0.412, p < 0.001); each correlation plot is accompanied by the respective Bland-Altman plot (red line: mean difference of two measures. Green lines: mean difference of two measures ± 1.96 x standard deviation).

A patient example applying the best threshold of the different approaches on a single LNM is shown in **Figure 1**. For visualization of the association of the best threshold of the different approaches with the reference standard, correlation plots and the respective Bland-Altman plots are shown in **Figures 2** and **3**.

### PSMA-Avidity of Background Tissues

Highest median SUV_mean_ in background tissues was found in the parotid gland followed by the liver and spleen (lowest uptake), i. e. 20.1 (range, 5.8 - 36.3) *vs.* 11.3 (range, 4.2 - 25.5) *vs.* 9.9 (4.7 – 28.7), p<0.001. These uptake values lead to an CoV of 42.6% using SUV_spleen_, followed by 40.2% using SUV_liver_ and the lowest CoV of 35.6% using SUV_parotis_. PSMA-avidity of background tissues (SUV_liver_, SUV_parotis_ & SUV_spleen_) did not show a significant correlation with each other (p>0.05 each) (please see **Table 4**).

### Individual Backwards Thresholding

On an individual, single lymph node basis, threshold values were individually adjusted in order to achieve the very same PET-based volume compared to the CT-based reference standard in each lymph node using a fixed SUV value, as this approach performed best in previous analyses. Here, the same volume compared to the CT-based reference was achieved using a mean SUV of 5.4 ± 2.4, which resulted in a high CoV of 44.4% among the fifty LNM. However, applying these resulting mean values of backwards thresholding to all 50 lymph nodes and correlating these volumes the CT-based volumetric reference (i. e. SUV 5.4 in all 50 lymph nodes), the correlation coefficient was inferior to previous analyses (i. e. r=0.764, r^2^ = 0.584, p<0.001) (see **Table 5**).

## Discussion

Measuring the volumetric extent of metastatic spread in prostate cancer is of fundamental interest in patients undergoing systemic therapy such as chemotherapy or radioligand therapy (17, 29) with potential impact on clinical decision making (7, 9, 30, 31). Due to its many advantages over ^68^Ga-labeled ligands, ^18^F-labeled compounds such as ^18^F-PSMA-1007 are becoming increasingly important for staging as well as treatment response assessment; in this analysis, we correlated tumor volumes derived from different threshold-based approaches for PET-based delineation with the CT-based, volumetric reference, i. e. the morphological volume of distinct, non-bulky lymph node metastases as derived from hybrid imaging using ^18^F-PSMA-1007 PET/CT.

Even if bone metastases present a common and clinically relevant metastatic spread in PCa patients (23), they are difficult to delineate on CT resulting in non-measurable lesions according to routine response criteria RECIST 1.1 (24, 25). Also, visceral metastases or lung metastases represent an unideal volumetric reference standard for the current issue, especially due to motion artefacts on PET/CT and unequivocal protocols concerning breath-holding impacting PET imaging. In contrast, LNM represent measurable metastatic sites, especially in case of large extent and non-bulky localization and were primarily evaluated in the current analysis.

In consideration of our results, we can state that a simple fixed SUV of 4.0 as threshold for tumor delineation without reference tissue correlated best with the volumetric reference standard (r=0.807, r^2 =^ 0.651, p<0.001) even though some of our acquired threshold values also showed comparable, but slightly lower correlation coefficients to the reference standard [e.g. 60% SUV_liver_ (r=0.715, r^2^ = 0.511, p<0.001) or 80% SUV_parotis_ (r=0.762, r^2^ = 0.581, p<0.001)]. These data are additionally supported by the visual analyses of the respective Bland-Altman plots (see **Figure 2**), where the approach using SUV 4.0 as delineation method also performed best.

Previously published optimized thresholds for intraprostatic tumor delineation on ^18^F-PSMA-1007 PET/CT (20%, 42% and 44% isocontour relative to SUV_max_) showed distinctly lower correlation to the reference standard compared to a fixed SUV of 4.0 (20% SUV%: r=0.460, r^2^ = 0.212. 42% SUV%: r=0.530, r^2^ = 0.28. 44% SUV%: r=0.552, r^2^ = 0.305, p<0.001 each), which indicates that these values seem feasible for delineation of the primary site of prostate cancer, but seem less feasible for delineation of lymph node volumes or even WTV in metastatic prostate cancer patients (22).

Obviously, it can be stated that the identification of the “one” ideal threshold value is a merely impossible task, as, on a cellular level, not all tumor cells can be delineated and be included in the image-derived WTV. However, a uniformly applied approach for PET-based delineation with the nearest approximation to a reference standard might, consequently, also allow a uniform and cross-institutional estimation of a WTV. We identified a simple SUV value of 4.0 as the threshold with the best correlation to the reference standard derived from large LNM. Thresholding using mere SUV values comprises several advantages: no specific software or algorithms are needed to determine WTV on ^18^F-PSMA-1007 PET/CT, as SUV is a commonly displayed unit in PET imaging. Moreover, no background/reference tissues are needed for WTV estimation making this analysis independent of potential change in PSMA-avidity in the reference tissues potentially changing over time or during systemic therapy, e.g. during ^177^Lu- or ^225^Ac-PSMA-radioligandtherapy (32, 33). Of note, we could show that on an inter-individual basis, the most commonly applied reference tissues (i.e. liver, parotis, spleen) do have a high inter-individual variability with CoV values up to 43%. Moreover, the respective PSMA-avidity of all three reference tissues is not correlated with one another on an intra-individual level, so that a general, uniform PSMA-avidity among healthy organs seems unlikely. These findings also support the application of a simple SUV-based approach without reference tissue.

When trying to derive an optimal threshold on a backwards step approach, i.e., setting the threshold value to achieve the same volume on PET in every single lymph node, one can state that the reverse deduction of a PET-based threshold is partially limited by the obtained dispersion of threshold-values, i.e., we observed an CoV of around 40% among the resulting threshold values. When directly applying the derived mean SUV value to all lymph nodes and performing a correlation analysis with the CT-based reference standard, a good correlation to the volumetric reference standard was observed, which was, however, still inferior compared to the mere application of a SUV value of 4.0.

**Figure 4:**
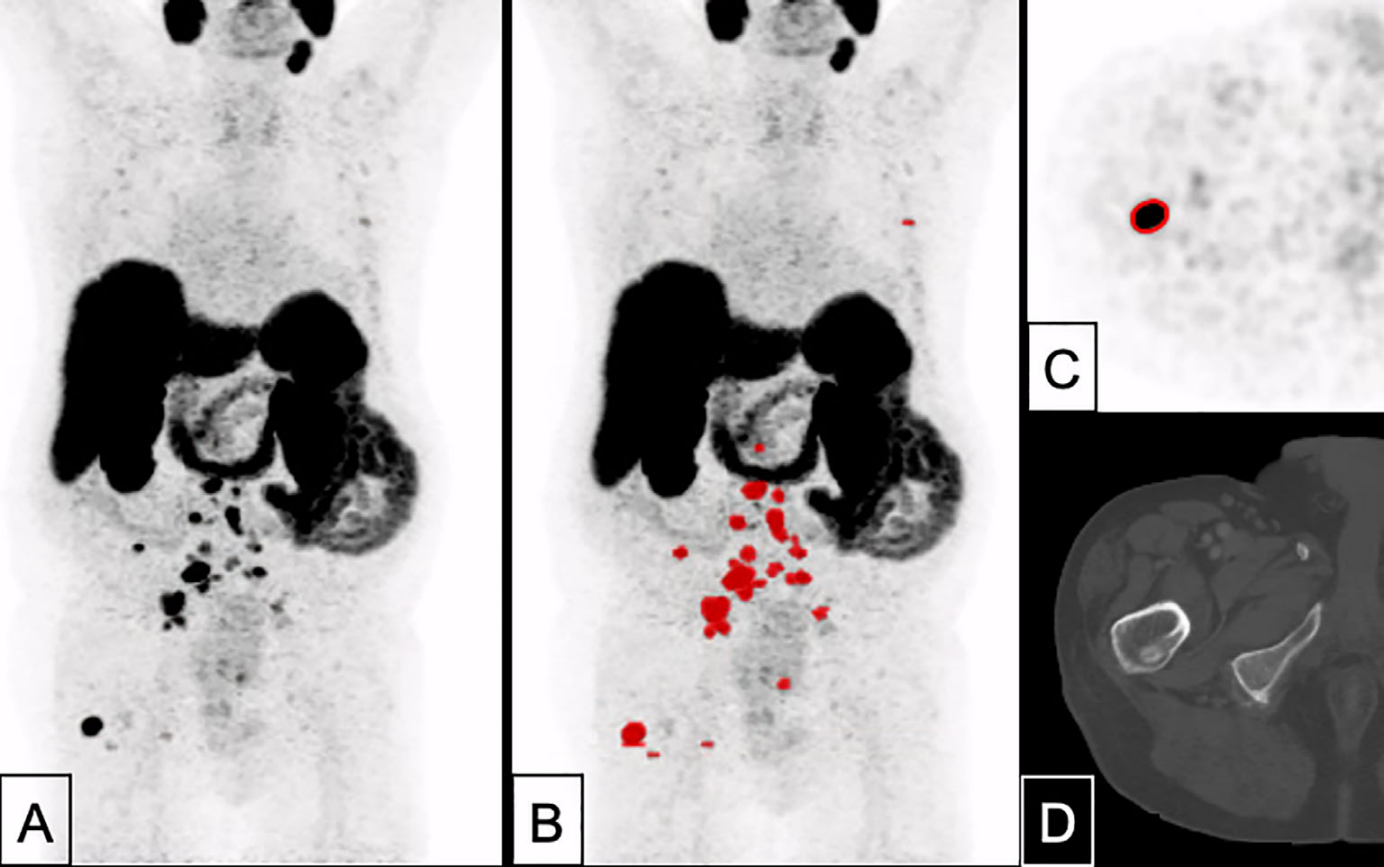
A 82 years-old patient with prostate cancer remnant as well as bone and lymph node metastases (PSA 10.1 ng/ml, Gleason 8). Tumor delineation using a cut-off of SUV 4.0 revealed a WTV of 37.9 ml. **(A)** maximum intensity projection (MIP); **(B)** MIP + WTV (red color); **(C)** delineation of a bone metastasis on PET; **(D)** CT correlate (bone window).

**Figure 5:**
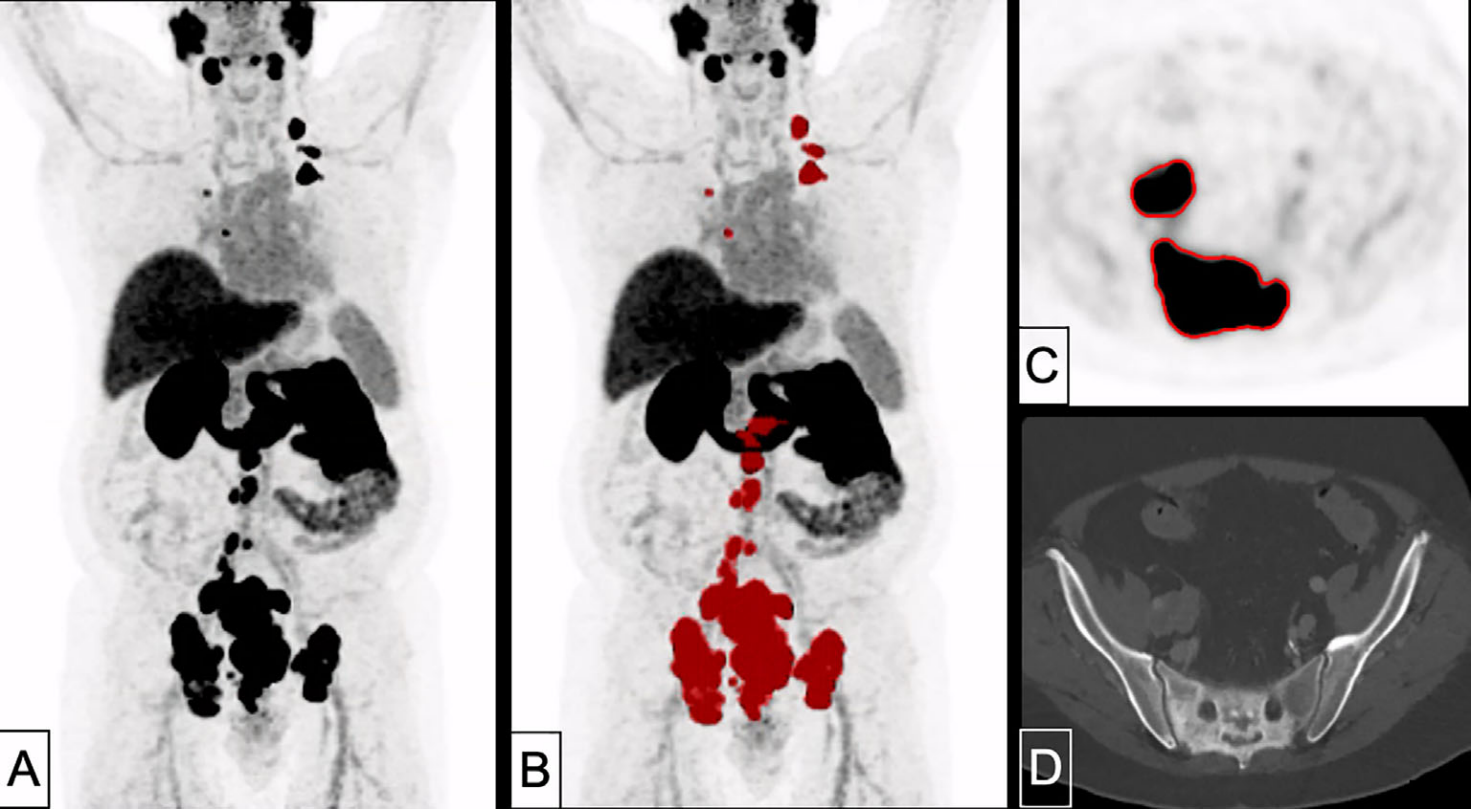
A 70 years-old patient with primary prostate cancer remnant with bone, pleura and lymph node metastases (PSA 78.0 ng/ml, Gleason 10). Tumor delineation using a cut-off of SUV 4.0 revealed a WTV of 586 ml. **(A)**: MIP; **(B)** MIP + WTV (red color); **(C)** delineation of bone and lymph node metastasis on PET; **(D)** CT correlate (bone window).

Overall, the application of a threshold of SUV 4.0 seems easily applicable in clinical routine, despite a certain blurriness regarding the actual nodal tumor volume. Given the partially extensive WTV in patients prior to systemic therapy, e.g., ^177^Lu-PSMA radioligand therapy, these small differences in lymph node volumes and small uncertainties in WTV do probably not carry a clinically relevant weight, when the same procedure is applied in a uniform manner consequently, so that the unavoidable blurriness is applied to all studies to the same degree. For potential translation of the derived threshold to other metastases, we included patient examples where the threshold of SUV 4.0 was applied for whole tumor volume delineation (see **Figures 4**, **5**) and showed a direct easy applicability and direct feasibility; nonetheless, further studies evaluating this threshold for WTV delineation and its course during therapy are the logical conclusion of the current analysis.

However, it has to be discussed that metastatic sites without significant PSMA-avidity (e.g. < SUV 4.0) are not included in the whole tumor volume as a consequence. In case of PSMA-negative, but clear metastatic spread on CT imaging (e.g. large bone metastases, bulky lymph nodes, etc.), but very low or even missing PSMA-avidity, a PSMA-derived tumor volume might underestimate the “real” tumor volume. Therefore, more specifically, the term “whole tumor volume” should be noted to be the “PSMA-avid whole tumor volume”. However, in the concrete case, if there are obvious metastatic sites on CT that are not included in the whole tumor volume due to very low or even missing PSMA-avidity, this fact should lead to e. g. an additional ^18^F-FDG PET for the evaluation of tumor dedifferentiation; in case of FDG-avid, non-PSMA-avid lesions, ^18^F-FDG PET imaging might be the superior modality for tumor characterization and, moreover, the application of PSMA-directed therapies should be critically discussed (34, 35).

Moreover, it should be noted that the application of this threshold potentially needs manual refinement, especially in case of close vicinity to areas or physiologically high PSMA-avidity such as the liver or guts, where the application of this threshold would cause a direct inclusion of lesions with physiological PSMA-avidity; however, this phenomenon is common for all PSMA-ligands and, moreover, also other ligands such as ^18^F-FDG, where areas of high glucose consumptions such as the brain do hamper automated lesion segmentation. E. g. in the rather rare case of liver metastases, the automatic delineation of liver metastases using this threshold SUV 4.0 has to be refined manually, especially, as the radioligand ^18^F-PSMA-1007 presents with a rather high biliary excretion (36). Nonetheless, in cases with liver metastases from prostate cancer, these cases usually present with generally high tumor burden so that small variabilities in manual refinement of liver metastases do not have a major impact on the absolute whole tumor volume. However, the issue of delineation of liver metastases is shared by nearly every PSMA-ligand in dependence of the particular degree of biliary excretion.

Moreover, using comparable PET/CT scanners from the same vendor with the same reconstruction algorithms and EARL accreditation, we observed a higher rate of dispersion regarding tumor delineation based on approaches relating to SUV_max_ as reference value, i. e. isocontour delineation. Our proposed delineation method, however, is based on a mere application of SUV values independent of the specific SUV_max_ value within metastatic sites. As also shown for other ligands (37), diverging PET-scanners and reconstruction algorithms do rather affect the reproducibility of SUV_max_ values than significantly lower, mere SUV values within the lesion. Therefore, the proposed delineation method should be more robust and reproducible compared to delineation methods relating to SUV_max_, as it seems less susceptible to diverging vendors and reconstruction algorithms. Further studies, however, have to address the reproducibility of PET parameters on ^18^F-PSMA-1007 PET in prostate cancer patients with emphasis on vendors and reconstruction algorithms beyond the scope of the current analysis.

Our analysis has several limitations that need to be considered: Some of the examined lymph nodes might potentially be susceptible to partial volume effect and spillover effects, even though we have chosen lymph nodes with a SAD of at least 1.0 cm (38). Another limitation is the retrospective design of the study as well as the fact that some of the lymph nodes were not histologically proven to be prostate cancer metastases. Nonetheless, our patients were already diagnosed with prostate cancer and presented with significantly increased PSA values and a high PSMA-expression of the lymph nodes, making an unspecifically high PSMA-avidity very unlikely. Moreover, readers were aware of common pitfalls with regard to lymph node detection, such as the presence of ganglia (39). In the future, a larger assessment with more patients is warranted to confirm our preliminary results. Additionally, further studies applying our approaches for total tumor volume delineation have to be performed to support our findings. Therefore, the concrete applicability of the currently derived threshold for metastatic sites other than lymph nodes has to be assessed systematically and has to be validated in the specific scenario of therapy monitoring of systemic treatments with assessment of WTV changes over time.

## Conclusions

A simple threshold of SUV 4.0 for delineation of nodal PCa lesions showed highest association with the volumetric reference standard independent of potential changes of PSMA-avidity in background tissues (e.g. parotis). This approach is easily applicable in clinical routine without specific software requirements. Further studies applying this approach for total tumor volume delineation are underway.

## Data Availability Statement

The original contributions presented in the study are included in the article/**Supplementary Material**, further inquiries can be directed to the corresponding author/s.

## Ethics Statement

The studies involving human participants were reviewed and approved by Ethics Committee, LMU Munich. Written informed consent for participation was not required for this study in accordance with the national legislation and the institutional requirements.

## Author Contributions

Manuscript draft/concept: LM and MU. Clinical management: NA, AT, MZ, VW, AH, AK, SL, and N-SS-H. Image analyses: LM, WK, HI, and MU. Supervision JR and PB. All authors increased the intelectual content of the work. All authors contributed to the article and approved the submitted version.

## Conflict of Interest

The authors declare that the research was conducted in the absence of any commercial or financial relationships that could be construed as a potential conflict of interest.

## Supplementary Material

Supplementary Table Extended patients’ characteristics.
