# Supplementary material for: Feasibility of Different Tumor Delineation Approaches for 18F-PSMA-1007 PET/CT Imaging in Prostate Cancer Patients
Source: Front Oncol. 2021 May 21;11:663631. doi: 10.3389/fonc.2021.663631 (PMC8176856; doi:10.3389/fonc.2021.663631)
Supplement: Supplementary Table — Extended patients’ characteristics. [file Table_1.docx]

|  | **Previous therapies** | | | | | | | **Current staging** | | | | | **Volumetric information** | | | | | | | |
| --- | --- | --- | --- | --- | --- | --- | --- | --- | --- | --- | --- | --- | --- | --- | --- | --- | --- | --- | --- | --- |
| **Patient #** | **Prosta-tectomy** | **Lympha-denectomy** | **Radio-**  **therapy** | **Anti**  **androgen therapy** | **Chemo-therapy** | **Xofigo** | **PSMA-RLT** | **PSA** | **Gleason Score** | **Tumor prostate bed** | **Bone metastases** | **Visceral metastases** | **SAD Lymph node** | **Volume lymph node** | **SUVmax lymph node** | **Volume SUV 4.0** | **55%SUVmax** | **60%SUVliver** | **80%SUVparotis** | **60%SUVspleen** |
| 1 | + | - | + | + | + | - | - | 19.2 | n.a. | - | + | - | 2.0 | 4.85 | 19.2 | 5.8 | 1.8 | 5.6 | 5.4 | 4.2 |
| 2 | - | - | + | + | + | - | - | 37.0 | n.a. | + | + | - | 2.0 | 6.30 | 28.4 | 5.5 | 1.0 | 4.5 | 6.4 | 4.0 |
| 3 | + | + | - | - | - | - | - | 8.5 | 9 | + | - | - | 2.0 | 5.27 | 35.4 | 7.3 | 1.2 | 5.5 | 8.4 | 5.5 |
| 4 | + | - | + | + | - | + | - | 34.8 | n.a. | - | + | + | 1.1 | 2.78 | 57.1 | 4.2 | 0.0 | 5.6 | 3.4 | 3.8 |
| 5 | - | + | + | + | + | - | - | 1118.0 | 9 | + | + | - | 1.2 | 2.12 | 20.2 | 3.1 | 0.8 | 5.5 | 6.7 | 5.9 |
| 6 | - | - | + | + | - | - | - | 127.0 | n.a. | + | + | + | 1.1 | 1.33 | 5.8 | 0.3 | 0.5 | 0.1 | 1.3 | 0.3 |
| 7 | + | + | + | + | + | - | - | 9.0 | 10 | - | + | - | 1.4 | 1.98 | 8.2 | 1.7 | 1.3 | 3.0 | 1.0 | 2.1 |
| 8 | + | - | + | + | - | - | - | 4.1 | 7 | - | - | - | 2.2 | 5.23 | 24.0 | 6.1 | 1.3 | 5.6 | 6.0 | 2.2 |
| 9 | - | - | - | - | - | - | - | n.a. | n.a. | + | + | - | 2.2 | 3.54 | 13.8 | 3.5 | 1.2 | 3.5 | 5.3 | 7.9 |
| 10 | - | + | + | + | - | - | - | 29.2 | n.a. | + | - | - | 1.7 | 1.14 | 12.8 | 2.7 | 1.6 | 0.4 | 4.5 | 3.0 |
| 11 | - | - | + | + | + | - | - | 35.0 | n.a. | + | + | - | 2.2 | 6.18 | 28.4 | 5.7 | 1.0 | 4.3 | 5.0 | 5.8 |
| 12 | - | - | - | + | + | - | - | 78.0 | 10 | + | + | + | 1.3 | 4.07 | 26.9 | 5.3 | 1.5 | 6.0 | 4.3 | 9.1 |
| 13 | + | + | + | + | - | - | - | 10.5 | 8 | - | - | - | 1.0 | 1.56 | 10.4 | 1.7 | 0.8 | 0.3 | 1.0 | 2.3 |
| 14 | - | - | - | n.a. | n.a. | n.a. | - | 12.8 | n.a. | + | - | - | 2.3 | 9.65 | 28.3 | 7.9 | 1.9 | 7.4 | 8.0 | 8.0 |
| 15 | + | - | - | + | + | - | - | 98.8 | 9 | - | + | - | 1.0 | 2.97 | 86.7 | 7.0 | 0.3 | 4.1 | 4.2 | 15.4 |
| 16 | - | - | - | + | - | - | - | 23.5 | n.a. | + | + | - | 2,7 | 23.77 | 59.3 | 26.3 | 2.7 | 23.8 | 25.0 | 26.0 |
| 17 | + | - | - | - | - | - | - | 2.5 | n.a. | - | - | - | 1.0 | 1.04 | 13.9 | 1.9 | 0.7 | 1.3 | 1.7 | 2.8 |
| 18 | - | - | - | + | - | - | - | 14.4 | n.a. | + | - | - | 1,2 | 1.09 | 6.0 | 0.7 | 1.1 | 0.0 | 0.0 | 0.4 |
| 19 | - | - | - | - | - | - | - | 6.0 | 9 | + | + | - | 2.0 | 5.00 | 27.1 | 6.2 | 3.1 | 4.2 | 6.7 | 7.3 |
| 20 | - | - | + | + | - | - | - | 70.4 | n.a. | + | + | - | 1.0 | 1.04 | 27.0 | 3.1 | 0.4 | 2.8 | 2.0 | 2.2 |
| 21 | + | + | - | - | - | - | - | 28.0 | 7 | - | - | - | 1.4 | 3.58 | 26.3 | 7.1 | 2.0 | 4.5 | 7.0 | 6.3 |
| 22 | - | - | + | + | + | - | + | 40,1 | n.a. | + | + | + | 1.3 | 4.08 | 6.3 | 2.3 | 1.2 | 2.6 | 5.2 | 3.9 |
| 23 | - | - | - | + | - | - | - | 7.5 | n.a. | + | + | - | 1.7 | 1.23 | 28.7 | 2.6 | 0.8 | 1.9 | 3.0 | 0.4 |
| 24 | - | - | + | + | + | - | - | 79.3 | 9 | - | + | - | 1.1 | 2.68 | 29.4 | 5.7 | 0.7 | 24.3 | 5.0 | 21.3 |
| 25 | - | - | - | - | - | - | - | 9.1 | 9 | + | + | - | 1.0 | 1.65 | 11.3 | 2.0 | 0.9 | 0.8 | 1.4 | 2.2 |
| 26 | - | - | + | + | - | - | - | n.a. | 9 | - | + | + | 1.0 | 2.62 | 8.8 | 2.6 | 1.7 | 0.5 | 1.8 | 3.1 |
| 27 | - | - | + | + | + | - | - | 1.2 | n.a. | + | + | - | 1.2 | 1.88 | 11.9 | 0.9 | 0.5 | 1.6 | 0.4 | 0.6 |
| 28 | - | - | - | + | - | - | - | 40.9 | 9 | + | + | + | 1.3 | 3.71 | 7.4 | 1.4 | 1.3 | 1.5 | 4.8 | 1.2 |
| 29 | - | - | - | - | - | - | - | 68.0 | 8 | + | + | - | 1.1 | 3.08 | 21.3 | 5.3 | 1.5 | 1.9 | 3.6 | 5.1 |
| 30 | + | - | - | + | - | - | - | 0.2 | n.a. | - | + | - | 1.7 | 10.41 | 14.9 | 6.0 | 1.2 | 5.0 | 7.4 | 2.9 |
| 31 | + | - | + | - | + | - | - | 2.3 | 9 | - | - | - | 2.1 | 18.86 | 8.7 | 12.4 | 8.4 | 16.8 | 13.3 | 18.6 |
| 32 | + | - | - | - | - | - | - | 0.6 | 9 | + | - | - | 2.8 | 15.04 | 14.3 | 16.9 | 8.5 | 15.3 | 13,8 | 17.5 |
| 33 | - | - | - | + | - | - | - | 10.1 | 8 | + | + | - | 1.5 | 6.47 | 16.9 | 8.3 | 2.1 | 8.1 | 6.9 | 5.8 |
| 34 | + | - | - | - | - | - | - | n.a. | n.a. | - | - | - | 1.2 | 2.00 | 10.0 | 1.8 | 1.1 | 0.1 | 1.8 | 1.7 |
| 35 | - | - | - | + | + | - | - | 2.1 | 9 | + | + | - | 1.2 | 4.77 | 55.5 | 3.9 | 0.2 | 5.4 | 3.6 | 2.9 |
| 36 | + | + | - | - | - | - | - | 2.3 | 9 | - | + | - | 1.4 | 2.70 | 10.2 | 2.0 | 0.9 | 1.9 | 0.5 | 2.0 |
| 37 | - | - | - | - | - | - | - | 80.0 | 9 | + | + | - | 1.2 | 1.59 | 8.4 | 1.6 | 0.9 | 0.0 | 2.6 | 3.0 |
| 38 | + | - | - | + | - | - | - | 48.6 | 6 | - | - | - | 1.1 | 2.26 | 15.0 | 6.9 | 2.3 | 4.3 | 7.6 | 5.4 |
| 39 | - | - | - | + | + | - | - | 331.0 | 9 | + | + | - | 1.7 | 7.16 | 29.1 | 10.1 | 1.9 | 9.8 | 10.6 | 10.2 |
| 40 | + | + | - | - | - | - | - | 13.9 | 8 | - | - | - | 1.4 | 1.84 | 6.7 | 0.7 | 1.0 | 1.6 | 0.0 | 0.5 |
| 41 | - | - | - | + | + | - | - | 4.7 | n.a. | + | + | - | 1.4 | 3.24 | 18.0 | 5.4 | 0.9 | 3.3 | 5.4 | 5.4 |
| 42 | + | + | + | + | - | - | + | 42.0 | 9 | - | + | + | 1.2 | 1.59 | 41.1 | 3.4 | 0.6 | 2.8 | 4.4 | 2.1 |
| 43 | - | - | - | + | - | - | + | 182.0 | n.a. | - | + | + | 1.2 | 1.61 | 10.2 | 2.3 | 0.7 | 2.3 | 2.2 | 2.5 |
| 44 | + | - | + | + | - | - | + | 441.0 | 8 | - | + | + | 1.3 | 4.10 | 38.1 | 9.0 | 1.6 | 6.5 | 24.7 | 6.3 |
| 45 | - | - | - | + | - | - | + | 1.2 | 6 | + | + | - | 2.8 | 3.23 | 13.5 | 3.6 | 1.2 | 2.1 | 5.7 | 7.5 |
| 46 | + | + | - | + | - | - | - | n.a. | n.a. | + | + | - | 1.7 | 4.83 | 15.1 | 4.3 | 0.8 | 2.0 | 3.2 | 12.3 |
| 47 | + | - | + | + | + | - | - | 40.0 | 9 | - | + | + | 1.4 | 1.92 | 39.0 | 4.7 | 0.8 | 6.6 | 9.7 | 5.5 |
| 48 | + | - | + | + | - | - | - | 13.0 | 7 | - | - | - | 1.6 | 14.58 | 38.7 | 19.0 | 2.5 | 20.7 | 12.5 | 18.4 |
| 49 | - | - | - | + | - | - | + | 447.0 | 8 | + | + | - | 1.1 | 2.14 | 38.7 | 4.5 | 0.9 | 11.8 | 4.4 | 7.7 |
| 50 | + | + | + | + | + | - | - | 588.0 | 8 | - | + | + | 1.1 | 8.42 | 16.7 | 9.1 | 1.7 | 11.6 | 12.1 | 5.4 |

**Supplementary Table**: Extended patients’ characteristics
